# GHG displacement factors of harvested wood products: the myth of substitution

**DOI:** 10.1038/s41598-020-77527-8

**Published:** 2020-11-27

**Authors:** Philippe Leturcq

**Affiliations:** University of Toulouse, Toulouse, France

**Keywords:** Climate sciences, Environmental sciences

## Abstract

A common idea is that substituting wood for fossil fuels and energy intensive materials is a better strategy in mitigating climate change than storing more carbon in forests. This opinion remains highly questionable for at least two reasons. Firstly, the carbon footprints of wood-products are underestimated as far as the “biomass carbon neutrality” assumption is involved in their determination, as it is often the case. When taking into account the forest carbon dynamics consecutive to wood harvest, and the limited lifetime of products, these carbon footprints are time-dependent and their presumed values under the carbon neutrality assumption are achieved only in steady-state conditions. Secondly, even if carbon footprints are correctly assessed, the benefit of substitutions is overestimated when all or parts of the wood products are supposed to replace non-wood products whatever the market conditions. Indeed, substitutions are effective only if an increase in wood product consumption implies verifiably a global reduction in non-wood productions. When these flaws in the evaluation of wood substitution effects are avoided, one must conclude that increased harvesting and wood utilization may be counter-productive for climate change mitigation objectives, especially when wood is used as a fuel.

## Introduction

A commonly held idea is that substituting wood for fossil fuels or energy intensive materials reduces greenhouse gas (GHG) emissions^1–8^. This opinion is supported by the values that are usually attributed to the “displacement factors” (DFs) which relate the emission reduction to the carbon mass contained in the wood used. When substituting a wood product for a functionally equivalent non-wood product, the displacement factor is defined, in accordance with common sense, by the following expression:

1 $$DF=(\left|{f}_{nw}\right|-\left|{f}_{w}\right|)/ {C}_{w}$$

where $\left|{f}_{nw}\right|$ and $\left|{f}_{w}\right|$ are the greenhouse gas emissions resulting from the use of the non-wood and the wood alternatives, respectively, expressed in mass units of carbon equivalent, and ${C}_{w}$ is the carbon mass content of the wood product. This definition follows that given by Sathre and O’Connor^9^, using simpler symbols and assuming there is no wood in the substituted product. ${f}_{w}$ and ${f}_{nw}$ identify with the carbon footprints of the wood and non-wood products, respectively. The displacement factor is dimensionless and applies whatever the quantity of products considered. When dealing with a set of exchangeable products, the overall DF is obtained by weighted average of the individual DFs.

However, the concept of displacement factor (or “substitution coefficient”) is frequently misused in practice. Notably, the DF values reported in the literature are questionable due to excessively simplifying assumptions in the evaluation of the carbon footprints of harvested wood products (HWP). The danger is that overestimated GHG emission reductions by substitution are an incentive to increasing wood harvesting, which could be counter-productive for climate change mitigation objectives and detrimental for forest ecosystems. Two critical flaws must be pointed out.

Firstly, the values that are usually attributed to the carbon footprints in definition (1) are most often based on the “carbon neutrality” assumption for wood and thus relate to the fossil carbon components of the total emissions only. “Carbon neutrality” refers to the supposition that, in sustainably managed forest-wood chains, the biogenic CO_2_ emissions coming from combustion or decomposition of wood are fully compensated, at all times, by the capture of atmospheric CO_2_ for tree growth. This supposition is unfounded in general, holding true only in static conditions, which are seldom met in practice. Furthermore, an additional harvesting implies a change in the forest carbon stock, thereby transiently suppressing this neutrality if it previously prevailed. Thus, “carbon neutrality” is now widely recognized as a misleading concept^10–14^. The carbon footprint evaluation of HWPs must include the components of biogenic carbon and these depend on the carbon dynamics in the forest domain from which the woody material originates^15–17^. Thus, if the $\left|{f}_{nw}\right|$ and $\left|{f}_{w}\right|$ terms are understood as measures for fossil emissions only, the corresponding DF value promises unrealistic GHG benefits^18–20^. Authors who are aware of this difficulty account separately carbon stock variations in forest and in wood-products^7,21–26^. Such an approach is valid as far as the interactions between the forest and wood-product carbon pools are correctly taken into account, but this does not remove the ambiguity of the displayed DF values. Another option, described in this article, is to return to the original definition of DF and to consider that the HWP carbon footprints include right away both biogenic and fossil emissions, as it is fundamentally the case.

Secondly, in assessing the climate change mitigation potential of forest growth and wood use at a regional, national or global scale, a current practice is to apply averaged displacement factors to large segments of the HWP production^27–32^. Most studies using this approach conclude that GHG benefits from substitution globally surpass those which would result from the growth of existing forests, thus inciting to increase harvesting. This conclusion is doubtful, even if biogenic emissions are correctly taken into account, if the DF values used are unweighted averages, or the set of HWP considered include products without non-wood equivalent, or the envisaged substitutions cannot be effective due to market conditions^33–35^.

Thus, effects of substitution, as they are commonly evaluated, do not correspond to reality and a back to basics is necessary.

## Basics

### Carbon exchanges between the “forest-wood” system and the atmosphere

**Figure 1:**
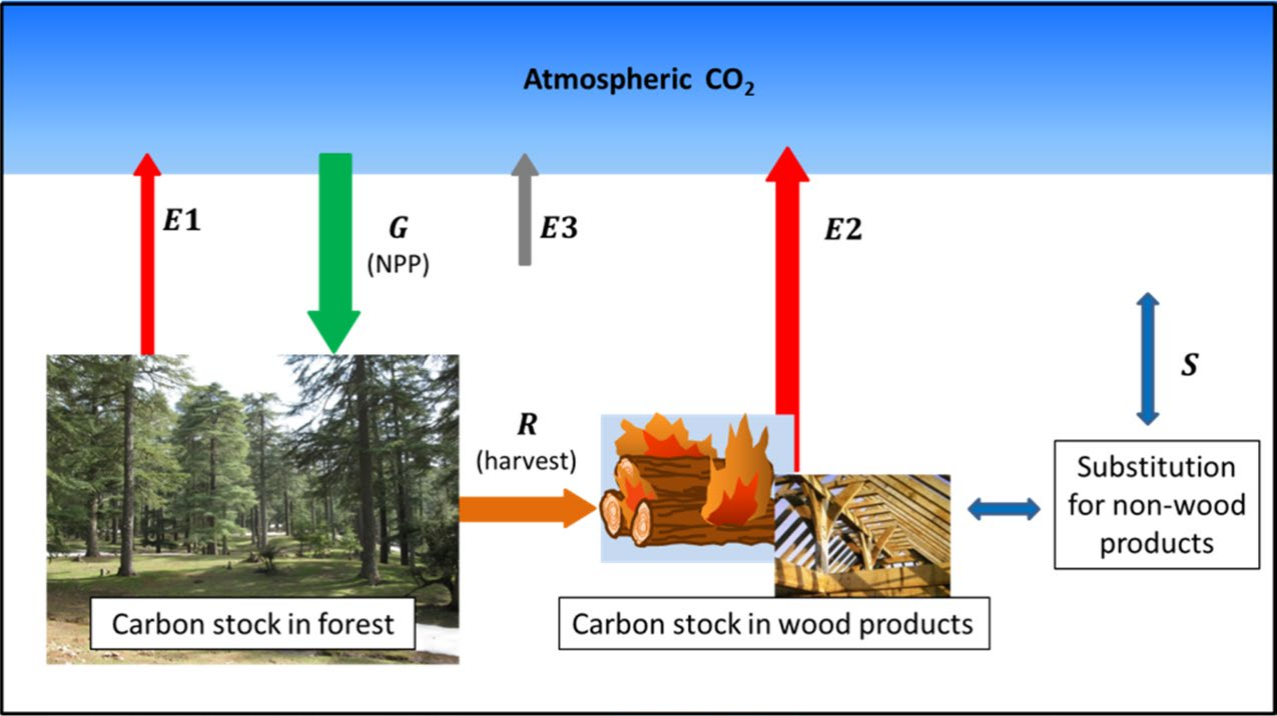
Carbon flux exchanges between forest, wood products and atmosphere (G: Net primary production; E1: decomposition of dead organic matter; R: harvested wood; E2: combustion or decomposition of harvested wood; E3: external emissions; S: substitution effect).

Figure 1 is a schematic description of carbon flux exchanges between the forest, the HWP sector and the atmosphere. The forest perimeter considered is arbitrary: it can be a single stand or the forest of an entire country. $G$ represents the net primary production (NPP) of biomass. $E1$ is the emission due to the decomposition of dead organic matter and $R$ is the amount of harvested wood. All these fluxes can be expressed in terms of carbon mass on an annual basis. $E2$ is the post-harvest carbon emission originating from the use of wood as a fuel and/or from the combustion or decomposition of woody residues and discarded products. $S$ indicates the emission variation resulting from selected substitutions of wood fuel or manufactured wood products for non-wood products. Disturbances such as wildfires or diseases may be considered through emission $E1$. An additional emission $E3$ includes the external fossil emissions that are linked to forest management, harvest, transport, industry processes, etc. Two carbon stocks must be considered: one in the forest biomass, the other in the wood products. The variations in these two stocks depend on the related carbon fluxes according to the mass conservation principle.

Leaving aside the additional $E3$ emission for the moment, the net primary production $G$ may be greater or less than the sum of $E1$ and $E2$ emissions. In the first case, the forest and the wood-product sector behave as a carbon sink for the atmosphere, allowing direct climate change mitigation. Conversely, in the second case, the “forest-wood system” acts as a carbon source, which should be avoided. There is “carbon neutrality” if $G$ equals the sum $E1+E2$, which can only be seen as a special case. In another way, harvested wood can be used to replace other fuels or other materials. Such a substitution can be favourable or unfavourable, depending on whether the life cycle of the displaced product is more or less emissive than that of the wood product. Therefore, two mitigation strategies are possible a priori: the one is direct and consists in reinforcing the carbon sink; the other is indirect and lies in an increase in wood harvesting with a view to favourable substitutions. These two strategies are opposite, since increasing the harvest cannot be done without weakening the forest carbon sink, unless concurrent afforestation.

Since the forest-wood system exchanges carbon only with the atmosphere, the carbon footprint of any action is represented, at every moment, by the consecutive change in the carbon stock of the system, in comparison with the carbon stock which would be observed in absence of this action (baseline). Referring to Fig. 1, the ”intrinsic” (or “biogenic”) carbon footprint of wood harvest and utilization cumulates the change in the forest carbon stock due to harvesting and that in the carbon stock of the products originating from the harvest. The additional (or “extrinsic”) $E3$ emission that is external to the forest-wood system can be assessed separately and added ultimately to the intrinsic carbon footprint.

### Carbon footprint of wood harvest

**Figure 2:**
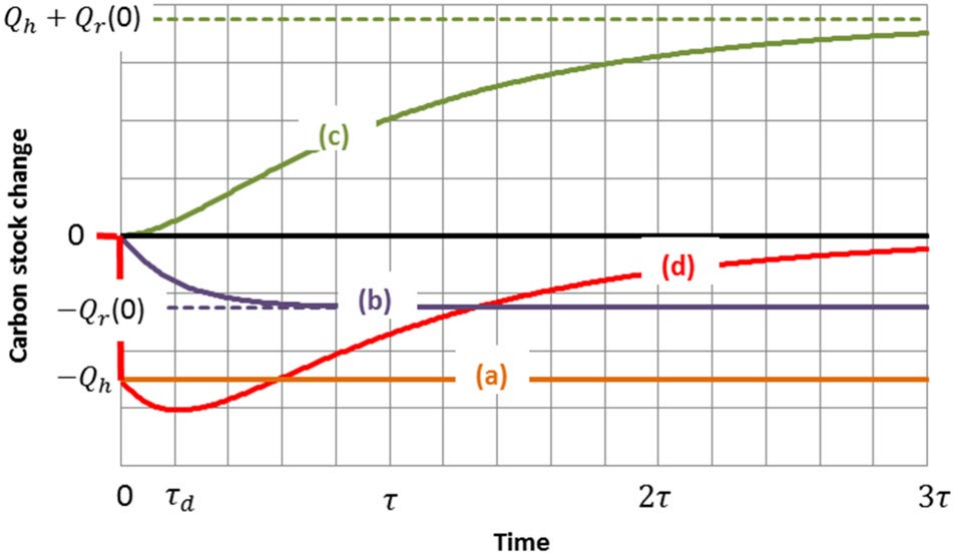
Main components of the forest carbon stock change (**d**) due to harvesting: (**a**): carbon content of the harvested wood (**b**): decomposition of harvest residues (**c**): forest regeneration.

Figure 2 illustrates the consequence of harvest on the forest carbon stock variation relative to that which would be observed without harvest. The simple case considered is a single harvest with a carbon content ${Q}_{h}$ operated in a forest containing a much larger stock. There is no loss of generality as far as the effects of sequential harvests are additive. Whatever the prior evolution of the forest carbon stock, increasing, decreasing or constancy, the consequence of harvest is the same. Three main processes act together. (a) The removal of ${Q}_{h}$ is a step change in the forest carbon stock. (b) Harvest residues that are principally worthless branches and tops, stumps, coarse roots and other debris may be left in place or partly removed from the forest, then being part of the harvested wood. Abandoned residues have a noticeable carbon content ${Q}_{r}(t)$ which may initially amount to as much as 50% of ${Q}_{h}$ if all debris are left on site. The carbon content ${Q}_{r}(t)$ continues to be a part of the forest stock but decreases progressively in time owing to decomposition of the residues. The decay has an exponential appearance following an approximate first order dynamics with a time constant ${\tau }_{d}$. (c) The forest regenerates, implying a positive change in carbon stock, which may be viewed approximately as exponentially shaped with a dominant time-constant τ. Under conditions that tree species, soil fertility, climate and forestry management remain unchanged, the same final equilibrium state of the forest can be expected to be reached whether or not harvest occurs, or, in other terms, the post-harvest evolution of the forest converges to that without harvest (base-line). In that case, the carbon stock total change (d), which represents the intrinsic carbon footprint of harvesting as regards the forest, would tend towards zero. Figure 2 is qualitatively constructed according to these considerations. The time-constant $\tau$ can be viewed as the mean residence time of carbon in forest biomass, in the absence of harvest, and serves as the time unit for the horizontal axis. Values of $\tau$ may range from a few decades for short rotation coppices up to several centuries for semi-natural or natural forests. In Fig. 2, the decomposition time constant of harvest residues ${\tau }_{d}$ is presumed smaller than $\tau$. The implicit sign convention is (−) for carbon emission, (+) for carbon capture.

As evidenced by Fig. 2, the intrinsic carbon footprint of harvesting, which coincides with the total carbon stock change (d), is formed over time and cannot be summed up in a single figure. According to the usual values of the main time constant $\tau$, the time axis scale is graduated in decades or even centuries and therefore may go far beyond the deadlines asserted by the United Nations Climate Change Conferences (e.g. 2050 and 2100). Hence the need to set time horizons compatible with these deadlines, such as what is done when comparing the global warming potentials (GWP) of different greenhouse gases. However, it should be pointed out that curve (c) is speculative since it depends on the future growth of the forest. Regeneration towards the same final state as without harvesting is only an assumption, even if it is often taken as a reference case in the literature. Other hypothesis may include changes in climate, tree species, forest management, and the possibility of disturbances. Thus, it should be kept in mind that the intrinsic carbon footprint is composed of two terms. The first one (negative) is the sum of the components (a) and (b) in Fig. 2 and can be fully determined, according to the IPCC guidelines for GHG inventories^36^ for instance. The second one (positive) corresponds to a regeneration term, exemplified by (c), which should be considered as conjectural. Nevertheless, for simplicity, we conserve the schema of Fig. 2 for the following illustrations.

## Results and discussions

### Carbon footprint of wood utilization as a fuel

**Figure 3:**
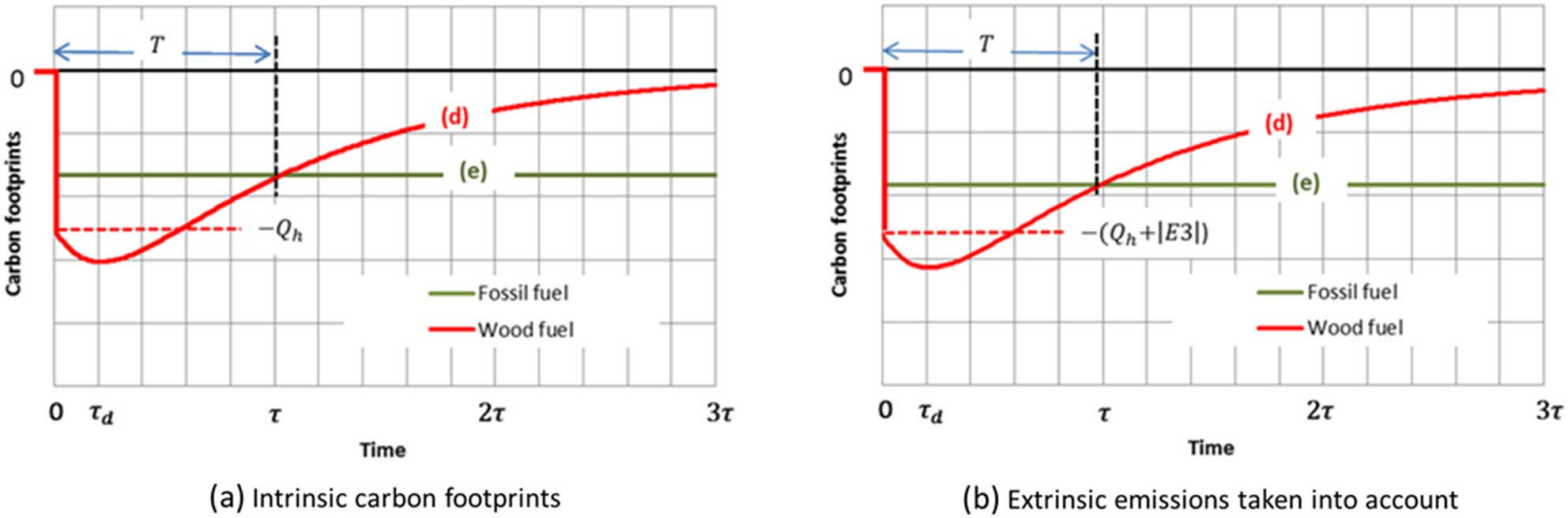
Comparison of the carbon footprints of wood and fossil fuels for the same energy released.

Line (d) in Fig. 2 also represents the intrinsic carbon footprint of the forest-wood system for an immediate use of the harvested wood as a fuel, since no carbon stock change occurs in the wood-product sector (see Fig. 3a). Soon after harvest (time $t$ much less than ${\tau }_{d}$ and $\tau$), the absolute value of the carbon footprint is not perceptibly different from the carbon content ${Q}_{h}$ of the wood fuel. This justifies a carbon accounting of common sense (but short-sighted) which considers that the wood that is burnt simply returns its carbon to the atmosphere. For times $t$ less than $\tau$ but more than ${\tau }_{d}$ (${\tau }_{d}$ is generally much less than $\tau$), the carbon of the harvest residues is added and, as far as the forest carbon stock recovery remains negligible, the absolute value of the footprint approaches the sum ${Q}_{h}+ {Q}_{r}(0)$. Afterwards, the variation of the carbon footprint is dictated mainly by the gradual recovery of the carbon mass ${Q}_{h}+ {Q}_{r}(0)$ as the forest regenerates. The carbon footprint thus goes through an extremum and finally, for $t\to \infty$, tends towards zero under the conditions specified above (and only under these conditions).

When comparing wood fuel to fossil fuels as regards climatic impacts, the pertinent physical characteristic is the CO_2_ emission factor, that is the amount of carbon (or carbon dioxide) emitted per unit of released energy. Values of this intrinsic emission factor taken from a standard source^37^ are shown in Table 1 (second column) for representative fuels. The combustion emission of wood is clearly higher than that of other fuels.

Therefore, as long as extrinsic emissions ($E3$ for wood) related to extraction, transportation, refining and distribution of fuels can be ignored or neglected, the carbon footprint of the use of a fossil fuel giving the same energy as that of harvested wood may be represented in Fig. 3a by a step function (e). The step amplitude is reduced, compared to ${Q}_{h}$, according to the ratio of the emission factor of the fossil fuel to that of wood. The use of wood as a fuel then appears to be intrinsically more emissive than that of other fuels for time horizons nearer than the time $T$ corresponding to the intersection of the lines that represent the carbon footprints in Fig. 3. The carbon footprint of using wood, on the other hand, is less (in absolute value) than that of the fossil fuel beyond $T$. Time $T$ corresponds to the “time to sequestration parity” according to the terminology of Mitchell et al.^16^.

Some complexity is added when one takes into account extrinsic emissions ($E3$ for wood) and also small quantities of GHG other than the CO_2_ produced in combustion. Typical values of the resulting effective emission factor^38^ in terms of CO_2_ equivalent are given in the third column of Table 1. Since the extrinsic emissions are small compared to the intrinsic ones, Fig. 3a must be only slightly modified (Fig. 3b).

Thus, as evidenced by Fig. 3, the displacement factor that quantifies the difference between the carbon footprint of wood and that of the substituted fuel is itself time-dependent. Just after combustion, displacement factors exhibit negative values, whatever the displaced fossil fuel^39^, as shown in the fourth column of Table 1. As the wood carbon footprint is increased transiently by a value in the order of the carbon content ${Q}_{r}(0)$ of the residues (extremum), the apparent wood emission factor is itself increased, reaching a peak up to 170 kgCO_2_eq/GJ or thereabouts if all crop residues are left in place. This estimate gives support to the rough affirmation that the carbon emission from wood fuel is bigger by half than that from coal, twice that from oil and three times that from gas, for the same energy released^40^. The consequent approximate DF values are displayed in the fifth column of Table 1. A positive value of DFs indicating a GHG benefit may be achieved only beyond the time $T$ to sequestration parity. Finally, long after harvest and under condition that the effect of harvesting on the forest carbon stock evolution totally vanishes, the wood energy may be seen as “carbon neutral”. The carbon footprint of the use of wood as a fuel is then reduced to the additional $E3$ emission. In this case, the displacement factors take the values indicated in the last column of Table 1.

**Table 1:** Typical emission factors for representative fuels^37,38^, and displacement factors by wood substitution.

Fuel	Intrinsic emission factor (kgCO_2_/GJ)	Effective emission factor (kgCO_2_eq/GJ)	Displacement factor (tC/tC)		
			At combustion	At extremum (approximately)	Under C neutrality assumption
Wood	112	117	–	–	–
Anthracite	98	103	**−** 0.12	**−** 0.62	+ 0.88
Heating oil	73	85	**−** 0.29	**−** 0.79	+ 0.71
Natural gas	56	67	**−** 0.45	**−** 0.95	+ 0.55

The effective emission factors are obtained by adding default values of extrinsic emissions to the intrinsic ones. Displacement factors are dimensionless (tC/tC or kgCO_2_/kgCO_2_). The values of displacement factors are calculated (i) at combustion, from the effective values of emission factors, (ii) at extremum of the carbon footprint of wood fuel (see Fig. 3), from a rough estimate of this extremum in the worst case where the carbon mass of the residues amounts to 50% of the carbon content of the harvest, (iii) under the carbon neutrality assumption, by setting 5 kgCO_2_eq/GJ (default value of E3) for the emission factor of wood.

Under the conditions of the explanatory Fig. 2, the values of $T$ are approximately $0.7\tau$ for a wood-for-coal substitution, $1.25\tau$ for a wood-for-gas substitution. $T$ nearly equals $\tau$ for a wood-for-oil substitution (case represented by Fig. 3). The expected actual values of $T$ may vary considerably depending on the forest carbon dynamics, substituted fossil fuels^41–43^ and also end-use technologies^19,42^. The efficiency of the end-use is generally lower for wood than for other fuels in thermal applications. By taking this efficiency into account, that is to say by referring to the unit of heat delivered by the fireplace, not to the unit of energy released at combustion, the differences between the effective emission factor of wood and those of other fuels would be slightly greater in relative value. These differences would be even wider in the case of electricity production, by reference to the unit of electric energy produced, due to a lower conversion efficiency of electric plants fed with wood. The sequestration parity time values would then be significantly increased in comparison with those determined by following the simple procedure exemplified by Fig. 3. But, above all, the time $T$ beyond which the GHG benefit of wood fuel use would become perceptible remains hypothetical, dependent on the forest regrowth. This time may be much greater than the preceding values, or even non-existent, if regeneration does not begin immediately after harvest (lack of capture) or is impeded by new conditions, climate change notably. Moreover, as coal may be advantageously displaced by oil, oil by natural gas and gas by non-carbonaceous sources of energy that are available, a GHG benefit from using wood as a fuel should soon be out from the field of possibilities.

### Carbon footprint of wood utilization as a material

**Figure 4:**
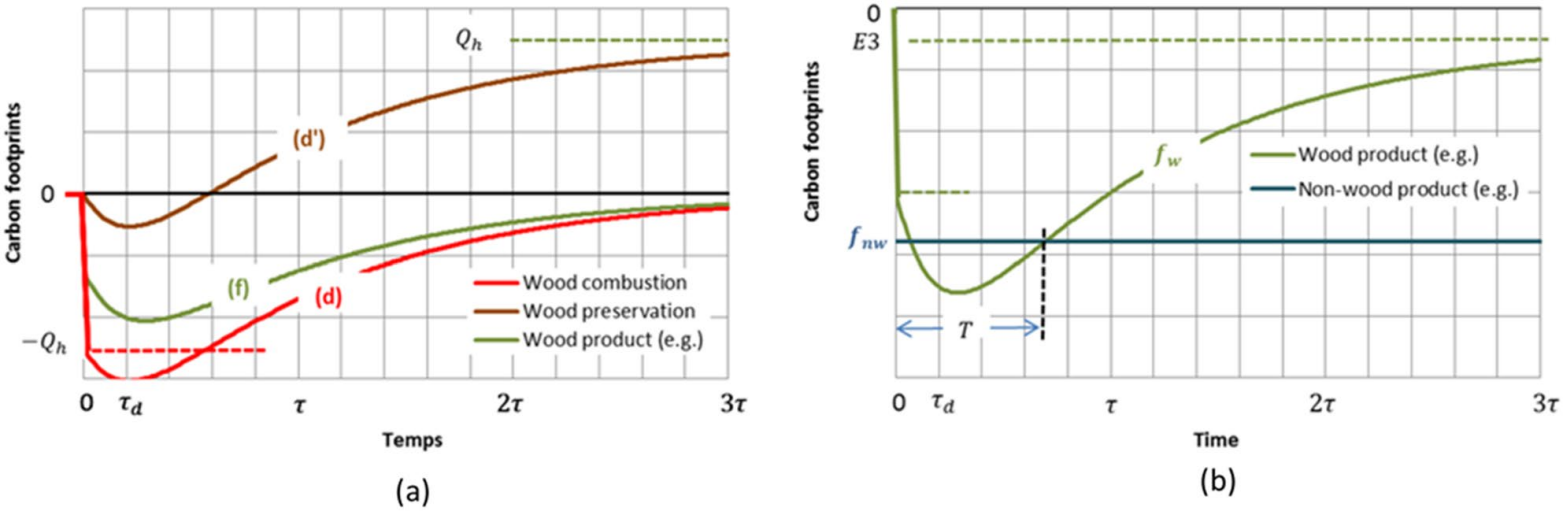
Example of a carbon footprint of a wood product (**a**) and definition of the time to sequestration parity in the substitution for a non-wood product (**b**). The vertical scale unit in (**b**) is divided in two for clarity in comparison to that in (**a**).

If the harvested wood were fully and sustainably preserved instead of being burned, its carbon content would be added to the forest stock. The carbon footprint variation then would be similar to that corresponding to the case of combustion, taking into account a positive shift of amplitude ${Q}_{h}$ along the ordinate axis (Fig. 4). Leaving aside the $E3$ emission which here also includes emissions linked to manufacturing processes, the two lines (d) and (d′) in Fig. 4a then limit the range in which the carbon footprint falls within, according to the proportion of woody residues in the industry processes and the lifespans of the manufactured wood products. The proportion of woody residues can amount to half, or more, of the raw material and the manufactured products become residues themselves when they reach the end of their use. These residues generally release their carbon, quickly through combustion, progressively through decomposition, so that the carbon footprint of the wood utilization as a material is, in most cases, closer to the lower limit (d) than the upper one (d′). Line (f) gives an example for a proportion of woody industrial residues of 50% of the raw material, and an average wood product lifespan comparable to the time constant $\tau$.

In order for the footprint to become positive (net capture), at least temporarily, most of the harvested wood material should therefore be preserved as long as possible. Nevertheless, barring particular options as burying wood^44^, the intrinsic carbon footprint of using wood as a material tends to zero after a long time ($t\to \infty$), due to the limited lifespan of the products and the woody material itself. Consequently, the full carbon footprint ${f}_{w}$ tends towards the additional emission $E3$ (Fig. 4b) and can only become transiently positive in exceptional cases. Thus, carbon storage in wood products generally has no direct mitigation effect on climate change.

The carbon footprint of substitutable non-wood products can be represented on graphs such as this of Fig. 4b by a horizontal line of ordinate ${f}_{nw}$, insofar as this footprint is fixed as soon as the products are manufactured. The time ranges for which the substitution of wood products for the non-wood products is GHG beneficial are bounded by the intersections of the two representative plots ${f}_{w}$ and ${f}_{nw}$. Thus, as for energy substitution, it is possible to define a time $T$ to sequestration parity beyond which a reduction of global emissions can be expected. If ${f}_{nw}$ is contained between the initial value and the extremum of the wood product footprint, there is also a short time window during which the substitution is temporarily beneficial. For higher values of $\left|{f}_{nw}\right|$ the substitution is permanently beneficial.

As Fig. 4b highlights, the displacement factor that quantifies the difference between the carbon footprints of the wood and non-wood alternatives is time dependent. According to definition (1), conjecturing a total recovery of woody biomass long after harvest and manufacture ($t\to \infty$), the displacement factor tends towards a constant value $(\left|{f}_{nw}\right|-\left|E3\right|)/{C}_{w}$. This is the value that is retained in studies that disregard changes in the forest carbon stock and refer to the assertion of carbon neutrality of wood. For instance, Sathre and O’Connor^9^ report displacement factors ranging from $-2.3$ to $15$ tons of carbon emission reduction per ton of carbon in wood products, depending on specific applications in building construction, with an unweighted average value of $2.1$ tC/tC. Many authors put forward comparable weighted estimates with slight differences owing to the country and application sector concerned^27–32^. However, as evidenced by Fig. 4b, the pertinent displacement factor values may be very different when definition (1) is applied at a definite moment or on average over a certain time horizon. When taking into account the decomposition of harvest residues (up to 50% of the harvest in carbon mass), the combustion or decomposition of the manufacture residues (typically between 50 and 70% of the harvest), the carbon footprint can peak, in addition to $\left|E3\right|$, to twice or four times the carbon mass in the wood product. Therefore, the displacement factor at the time when extremum occurs should be reduced by two or four units of tC/tC as compared to the above mentioned estimated values. The range of negative displacement factors for which substitution increases emissions instead of reducing them is then considerably broadened. In many cases, the GHG benefits expected from material substitution appear overestimated, as already inferred by some authors^45,46^. In addition, the scenario shown in Fig. 4 assumes that the forest regenerates completely after harvest. As in the case of the use of wood as fuel, such a scenario is only hypothetical, among others, and the very existence of time T is speculative.

### Applicability of the concept of substitution

Numerous studies have highlighted energy substitution and material substitution as forest levers for climate change mitigation^25,47–51^. Most authors consider that substitutable wood products may truly replace homologous non-wood products and apply averaged displacement factors to more or less extensive sets of wood products. This leads to estimates of emissions that would be globally “avoided” by substitution effects, at the scale of a region or an entire country. The amount of avoided emissions is thought of as enlarging the carbon sink of the forest-wood system. The results are an incentive for an increase in usage of wood as fuel or material and, upstream, for raising harvested wood volumes. However, these avoided emissions, already overestimated when the “carbon neutrality” of wood is invoked, generally remain largely potential. The reason can be explained as follows.

Let us consider two types of products having the same function and the same lifespan, one made of wood, the other made of another material. Let ${N}_{w}$ and ${N}_{nw}$ be the number of units annually produced of these products. The absolute value $\left|E\right|$ of the greenhouse gas emission linked to this production is:

2 $$\left|E\right|=\left|{f}_{w}\right|{N}_{w} + \left|{f}_{nw}\right|{N}_{nw}$$

where $\left|{f}_{w}\right|$ and $\left|{f}_{nw}\right|$ are the respective absolute values of the carbon footprints by units of these two types of products. If the productions ${N}_{w}$ and ${N}_{nw}$ are constant from year to year, the emission $\left|E\right|$ is itself constant and there is no reason to make one or the other production benefit from any "credit" of substitution. As specified by Sathre and O’Connor^52^, “*Taking a greenhouse gas “credit ” for wood substitution is only valid if the application of wood is verifiably a substitution for another material. There is no additional GHG benefit in the continued use of wood products for applications where they are typically already used”.*

There is an increase or reduction in emission only in relation to the annual variations ∆ of the productions ${N}_{w}$ and ${N}_{nw}$:

3 $$\Delta \left|E\right|=\left|{f}_{w}\right|\Delta {N}_{w} + \left|{f}_{nw}\right|\Delta {N}_{nw}$$

If we impose $\Delta {N}_{nw}=-\Delta {N}_{w}$, there is effectively substitution of the wood products for the competing non-wood products. The displacement factor (before normalisation to the carbon mass in the wood products) is, in this case:

4 $$DF=-\Delta \left|E\right|/\Delta {N}_{w}= \left|{f}_{nw}\right|-\left|{f}_{w}\right|$$

This factor is negative (increase in emission) or positive (reduction in emission) depending on whether $\left|{f}_{w}\right| > \left|{f}_{nw}\right|$ or $\left|{f}_{w}\right| < \left|{f}_{nw}\right|$. Thus, the substitution coefficients cannot apply to productions (or consumptions) but only to their variations. It should also be specified that the "eligible" products are those which are effectively substitutable for other non-wood products (graphic paper, for example, has no real "non-wood" equivalent: a substitution credit or debit should not be attributed to its production).

**Figure 5:**
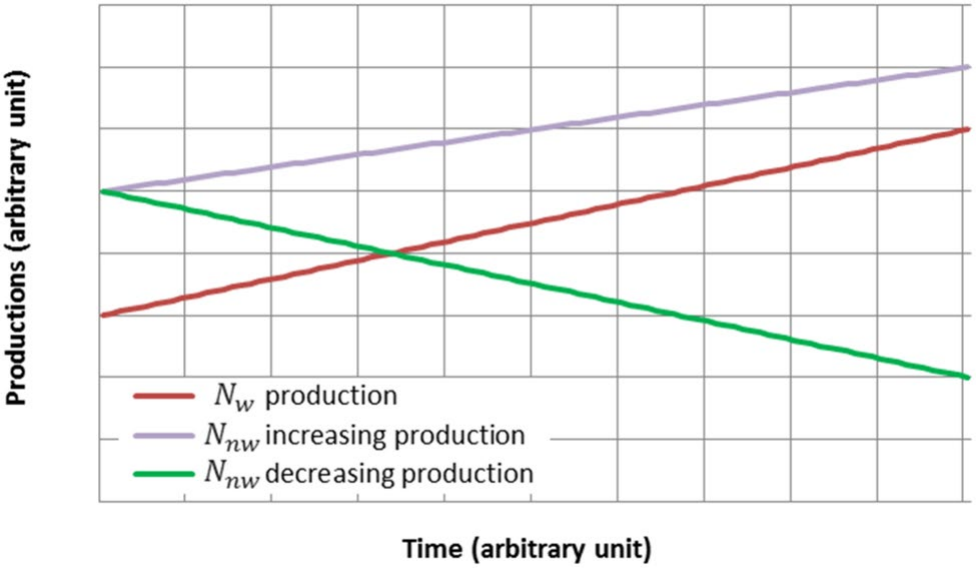
Examples of effective substitution (red line—green line) and simple variation of productions (red line—purple line).

Some authors^25,50^, who are aware of these conditions, restrict the application of displacement factors defined by Eq. (4) to increases in production of eligible wood products, compared to a reference year (marginal production). This restriction is necessary but is not sufficient. A critical example, inspired by publications by Déry^33^ and Bird^35^, is provided by the graphical representations in Fig. 5.

The production (or consumption) ${N}_{w}$ of the considered wood product is growing linearly in time (red line), while that ${N}_{nw}$ of the competing product is symmetrically decreasing (green line). The total production remains constant and $\Delta {N}_{nw}=-\Delta {N}_{w}$: there is effective substitution. Thus, according to Eq. (3):

5 $$\Delta \left|E\right|= \left(\left|{f}_{w}\right|-\left|{f}_{nw}\right|\right)\Delta {N}_{w}$$

In this case, the direct application of the displacement factor (4) to the variation $\Delta {N}_{w}$ is valid. However, let us suppose that the production of the alternative product also increases linearly (purple line) in response to a market demand, independently of that of the wood product. Then, the change in emission would just be:

6 $$\Delta \left|E\right|=\left|{f}_{w}\right| \Delta {N}_{w} + \left|{f}_{nw}\right|\Delta {N}_{nw}$$

This variation in emission cannot be attributed to one product rather than the other. Each of them has its share, $\left|{f}_{w}\right|\Delta {N}_{w}$ for the wood product, $\left|{f}_{nw}\right|\Delta {N}_{nw}$ for the competing product. There is no substitution but simply variation of the volumes produced and market shares. It would not be justified to attribute a credit or debit of emissions to one or the other of the productions.

A generalization is possible in the following way. If the total production variation $\Delta N$ is fixed a priori:

7 $$\Delta ({N}_{w}+{N}_{nw})=\Delta N$$

the variations of ${N}_{w}$ and ${N}_{nw}$ are then linked by $\Delta {N}_{nw}= \Delta N - \Delta {N}_{w}$. Consequently:

8 $$\Delta \left|E\right|=\left(\left|{f}_{w}\right|-\left|{f}_{nw}\right|\right) \Delta {N}_{w}+\left|{f}_{nw}\right|\Delta N$$

and the displacement factor to be applied is:

9 $$DF=-\Delta \left|E\right|/\Delta {N}_{w}= \left|{f}_{nw}\right|-\left|{f}_{w}\right|-\left|{f}_{nw}\right|\Delta N/\Delta {N}_{w}$$

expression which covers Eq. (4) for $\Delta N = 0$.

If there is no constraint on the variation of the total production, there is no reason to attribute substitution credits or debits.

## Conclusions

The substitution of wood for other fuels or other materials, which some believe to be more efficient in limiting the greenhouse effect than carbon capture and its direct storage in live trees, may be in reality counterproductive or, to say the least, its effects are greatly overestimated.

Energy substitution has an immediate adverse greenhouse effect since the emission factor of wood is higher than that of any other fuel. The possibility of regeneration for the exploited stands, and, in that case, the existence of a time delay beyond which a GHG benefit may be expected, cannot hide this inescapable physical reality. The time horizons set for the achievement of greenhouse gas emission reduction objectives (30 to 80 years) are, in most cases, less than this time to sequestration parity. It would therefore be appropriate to reduce the consumption of wood energy by the inverse substitution of less carbon intensive fuels or, better still, non-carbonaceous sources of energy.

Regarding material substitution, constant displacement factors put forward to promote it are overestimated, up to 2 to 4tC per ton of carbon for lumber products, for example, when referring to a near future. It is still possible to find favourable substitutions, particularly in the construction sector, but the total of the emission reductions can only be marginal, given that only effective substitutions can be taken into account.

The current option to increase forest harvesting with a view to climate change mitigation through substitution effects is therefore a serious error. This does not mean that the utilization of wood produced by forests is not legitimate, but only that this utilization cannot be justified by reasons of mitigation of climate change, except in very special cases. Forest exploitation and wood use should simply respond to technical, economic, social or societal needs while being subject, like other human activities, to a precise carbon accounting that allows judging, case by case, if they are well-founded (asserting “zero” for biomass emission factor is not an accounting practice).

Alternatively, to enable the forest to play an important and perhaps decisive role in mitigating climate change, the direct means of increasing wooded areas and standing tree volumes remain, therefore storing carbon in the forests and, under condition of GHG substitution benefits, in wood products. To be effective, this strategy must rely on reforestation and restoration of natural forests more than on the plantation of forests with productive objectives^53,54^.
